# Steel benchmark frames for structural analysis and validation studies: Finite element models and numerical simulation data

**DOI:** 10.1016/j.dib.2021.107564

**Published:** 2021-11-14

**Authors:** Constance W. Ziemian, Ronald D. Ziemian

**Affiliations:** a Department of Mechanical Engineering, Bucknell University, PA, Lewisburg, USA; b Department of Civil and Environmental Engineering, Bucknell University, PA, Lewisburg, USA

**Keywords:** Benchmark frames, Frame stability, Structural analysis, Numerical modelling

## Abstract

A sample of twenty-two steel benchmark frames was formed and used to investigate the accuracy of a single increment predictor-corrector (SIPC) solution scheme; an approximate method of second-order elastic analysis. Each of the frames is based on a structure from the literature and, as a collection, the set includes a diverse assortment of practical planar geometries and a wide range of sensitivities to second-order effects. This data article presents the details of these frames, including finite element models, relevant nodal coordinates and element connectivity, and detailed information regarding member sizes, support conditions, and applied loading. In addition, this article presents simulation data obtained from testing the SIPC method using the benchmark frames, and assessing its accuracy and precision. Error analysis results, based on comparisons of joint displacements and member design moments simulated using the SIPC method with those obtained using the more exact and computationally expensive work-control (WC) method, are summarized. The finite element modelling and subsequent structural analysis utilized the software package MASTAN2, which provides user-friendly features to execute both SIPC and WC methods. A detailed description of these analysis methods and the algorithms used to generate data is provided in “Efficient geometric nonlinear elastic analysis for design of steel structures: Benchmark studies” Ziemian and Ziemian [1]. The benchmark frame models are more generally useful for any researcher interested in testing and validating structural analysis and design methods, and the simulation data allow for comparisons with the results of other proposed solution schemes.

## Specifications Table

**Table:** 

Subject	Structural Engineering, Structural Stability
Specific subject area	Finite element analysis of steel benchmark frames; simulation of second-order elastic response to applied loading.
Type of data	Table Figure Graph MASTAN2 files (finite element models)
How data were acquired	Software: MASTAN2 (www.mastan2.com); Finite element models and numerical simulation of frame analysis Software: MATLAB (https://www.mathworks.com/products/matlab.html); Data error analysis
Data format	Raw; Source files: MASTAN2 Finite element models Filtered; Excel file: Nodal coordinates and FEM model information for each frame PDF file: Detailed frame information Analysed; Excel file: Results of SIPC and WC analyses
Parameters for data collection	Finite element models of benchmark frames were created using MASTAN2 structural analysis software. Frames were analysed using the proposed approximate method (Single Increment Predictor-Corrector or SIPC), the more exact work-control (WC) method, and a first-order linear analysis (LA) [1].
Description of data collection	The geometric nonlinear elastic response of each frame was simulated using SIPC and WC methods, by applying features provided within MASTAN2. Data comparisons and error analysis were performed using MATLAB.
Data source location	Bucknell University, Lewisburg, Pennsylvania, USA.
Data accessibility	Repository name: Mendeley Data Data identification number: https://doi.org/10.17632/39sjhchwtx.1 Direct URL to data: https://data.mendeley.com/datasets/39sjhchwtx/1
Related research article	C.W. Ziemian, R.D. Ziemian, (2021). Efficient geometric nonlinear elastic analysis for design of steel structures: Benchmark studies, *Journal of Constructional Steel Research*, 186, https://doi.org/10.1016/j.jcsr.2021.106870.

## Value of the Data

• The data provides a variety of useful resources for investigating structural behaviour and analysis methods using finite element software. The set of twenty-two benchmark frame models is important for testing/validating computational analysis methods and comparing results with those from commercial software and/or other works in the literature.
• Researchers and practicing engineers interested in studying or validating structural design and analysis methods and/or newly proposed solution schemes can use the developed set of benchmark frame models, which includes a variety of structural systems with a range of sensitivities to second order effects, realistic geometries, and boundary conditions that satisfy current design codes. Researchers and practicing engineers can also use the provided frame models/data and the associated analysis results to compare the performance of other proposed solution schemes to that of the SIPC method.
• The finite element models of the perfect and/or imperfect frames can be directly opened in MASTAN2 and further studied using any of the analyses and graphical visualization features available in the software. The frame nodal and connectivity data can be used to create the same benchmark structures (perfect and/or imperfect geometries) with other finite element software packages. The numerical simulation data can be used and/or reproduced as a basis for comparison with the results of other second-order elastic analysis methods or different approximate solution schemes.

## 1 Data Description

This data article presents the computational models and details associated with a collection of twenty-two planar steel frames, as well as the numerical simulation data produced by elastic analyses performed on the frames.

**Fig. 1:**
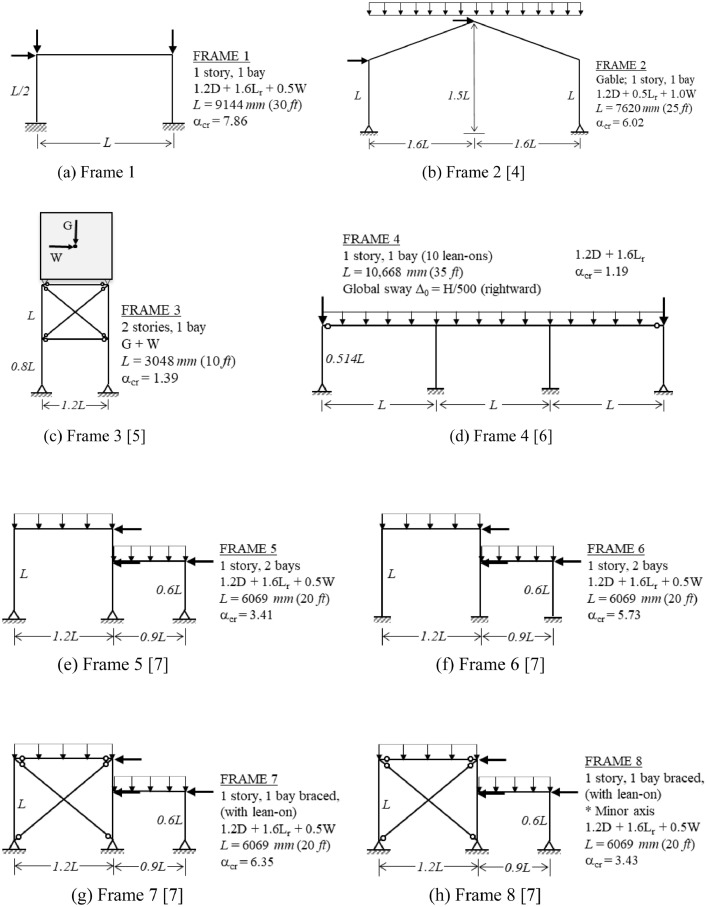

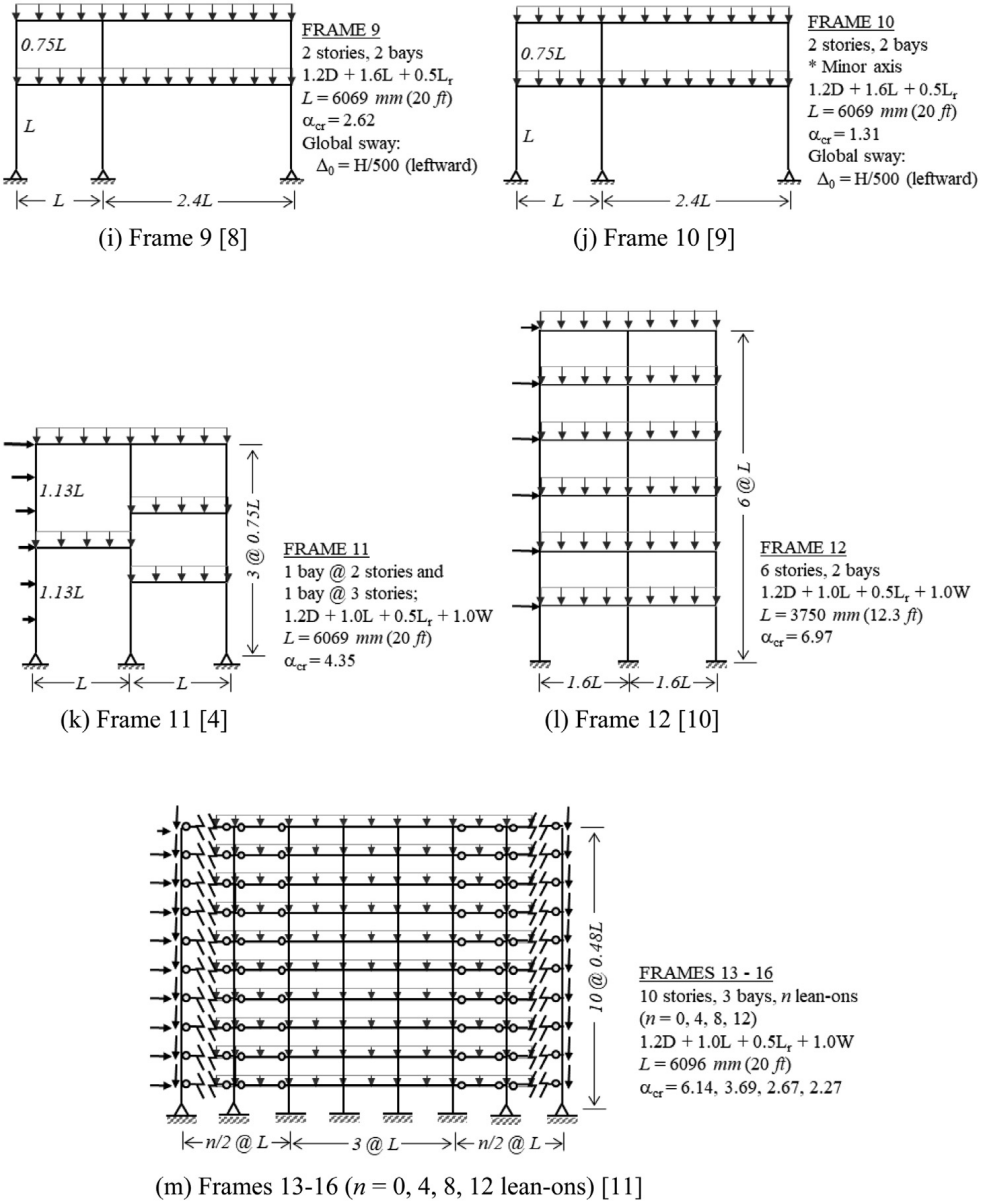

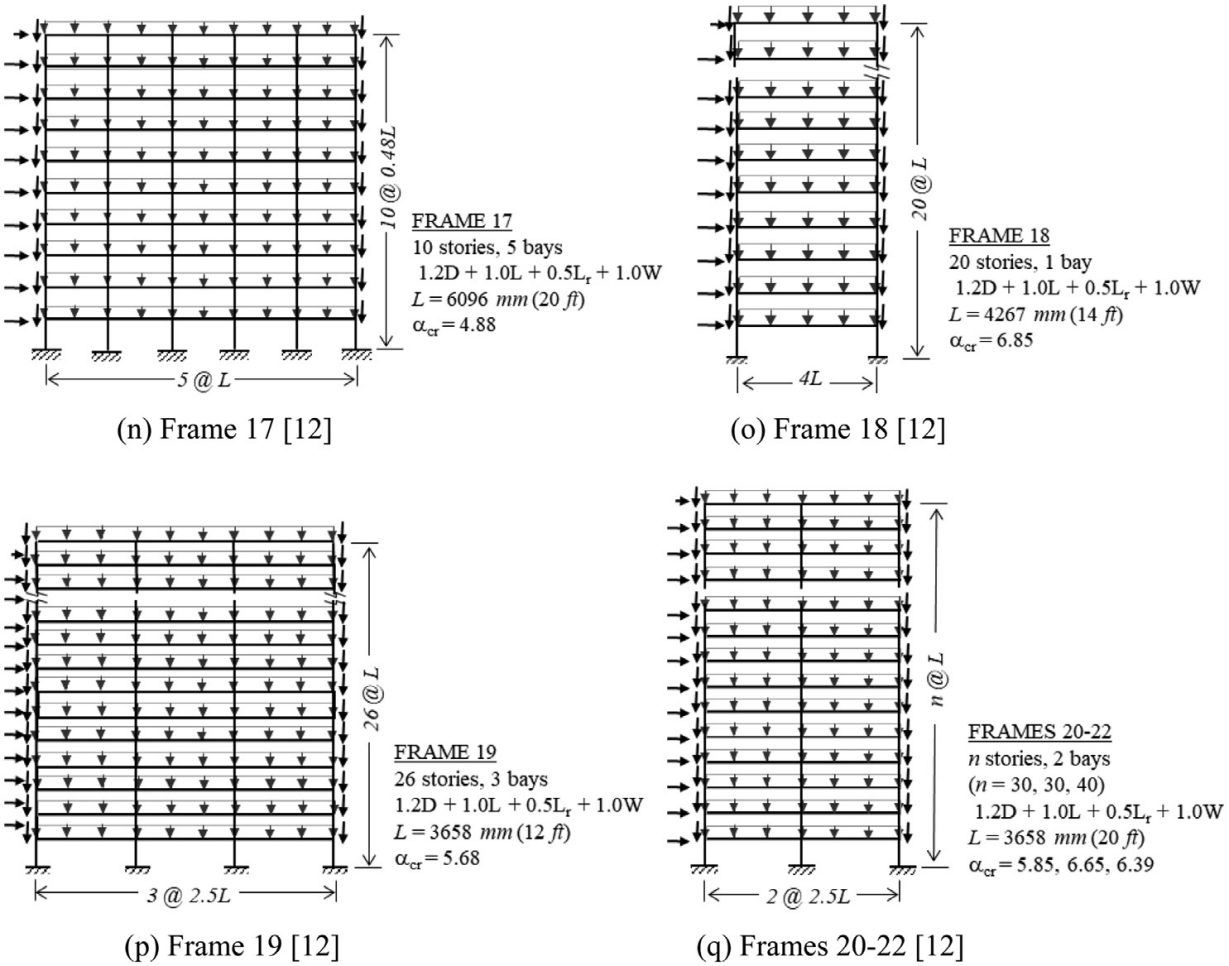
Overview of benchmark frames, including geometry, dimensions, load combination investigated, and critical buckling load ratio (α_cr_). Global imperfections are in the direction of lateral loading, unless otherwise noted. (a) Frame 1; (b) Frame 2 [4]; (c) Frame 3 [5]; (d) Frame 4 [6]; (e) Frame 5 [7]; (f) Frame 6 [7]; (g) Frame 7 [7]; (h) Frame 8 [7]; (i) Frame 9 [8]; (j) Frame 10 [9]; (k) Frame 11 [4]; (l) Frame 12 [10]; (m) Frames 13-16 (*n* = 0, 4, 8, 12 lean-ons) [11]; (n) Frame 17 [12]; (o) Frame 18 [12]; (p) Frame 19 [12]; (q) Frames 20-22 [12].

**Fig. 2:**
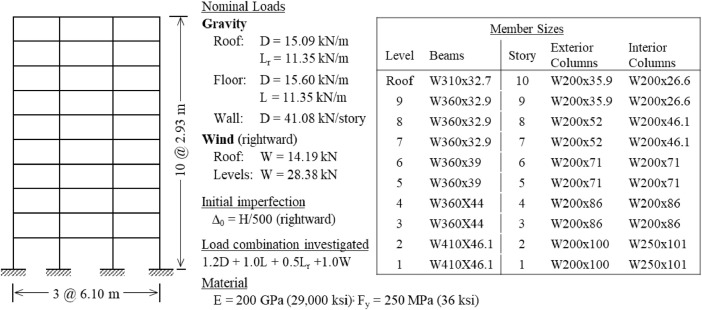
Sample of the detailed information available for each benchmark frame.
• Finite element models of both perfect and imperfect (with global sway) geometries (MASTAN2 [2] files).
• Finite element model data for both perfect and imperfect geometries (Excel file), including nodal coordinates and element connectivity, useful for creating the same frame models with other analysis programs.

The benchmark frames are introduced in Fig. 1, which displays the structure's geometry, load case investigated, and critical buckling load ratio α_cr_. For each frame in Fig. 1, the following data are provided in the Mendeley data repository [3]:

• Description of principal characteristics, including geometry, member sizes, and loading details (PDF file). A sample of this data is presented in Fig. 2.

Fig. 2 illustrates the detailed descriptive information that is provided as supplementary data [3] for each frame.

This set of benchmark frames has been used to test and validate a proposed solution scheme for the approximate second-order elastic analysis of structures; the single increment predictor-corrector (SIPC) method [1]. Table 1 presents a summary of the error analyses completed to assess the accuracy and precision of the SIPC method, including comparisons of simulation data produced using SIPC with those produced using the more exact and computationally expensive work-control (WC) method [13].

**Table 1:** Percent errors from comparisons of SIPC and WC results for benchmark frames (conservative (+) and un-conservative (-)). Frames sorted by indicators of stability sensitivity, $AF_{\alpha_{cr}}$ and $\left(\frac{\delta^{WC}}{\delta^{LA}}\right)$, from least to most sensitive.

Frames			Lateral joint displacements				Member design moments			
No.	AF_αcr_	$\frac{\delta^{WC}}{\delta^{LA}}$	Min	Median	Max	*Range*	Min	Median	Max	*Range*
1	1.14	1.15	−0.82	−0.82	−0.81	*0.01*	−0.83	−0.68	−0.68	*0.16*
12	1.16	1.17	−1.14	−1.12	−1.05	*0.09*	−1.20	−0.36	−0.01	*1.19*
18	1.15	1.17	−1.12	−1.04	−0.61	*0.51*	−0.90	−0.52	−0.02	*0.88*
21	1.17	1.18	−1.22	−1.18	−0.61	*0.61*	−1.33	−0.87	−0.04	*1.29*
22	1.18	1.19	−1.33	−1.23	−0.52	*0.82*	−1.56	−0.92	−0.07	*1.48*
7	1.18	1.19	−1.20	−0.71	−0.21	*1.00*	−1.04	−0.01	−0.01	*1.03*
13	1.17	1.19	−1.37	−1.27	−0.86	*0.51*	−1.59	−0.63	0.00	*1.59*
2	1.16	1.20	−1.64	−1.27	−1.24	*0.40*	−0.65	−0.63	−0.61	*0.04*
20	1.20	1.21	−1.60	−1.56	−0.88	*0.71*	−1.74	−1.03	−0.02	*1.71*
6	1.18	1.21	−1.38	−1.18	−0.97	*0.41*	−0.86	−0.25	0.01	*0.87*
19	1.20	1.21	−1.75	−1.70	−1.18	*0.57*	−1.89	−1.03	0.02	*1.91*
17	1.22	1.26	−2.35	−2.12	−1.67	*0.68*	−2.52	−0.91	0.03	*2.56*
11	1.24	1.30	−2.96	−2.73	−2.43	*0.53*	−2.35	−0.41	0.00	*2.35*
14	1.33	1.37	−4.08	−3.79	−2.47	*1.61*	−4.76	−2.19	−0.01	*4.75*
8	1.38	1.41	−4.54	−2.71	−0.88	*3.66*	−3.98	−0.01	−0.01	*3.97*
5	1.36	1.42	−5.50	−4.97	−4.49	*1.01*	−2.07	−0.82	−0.04	*2.03*
15	1.52	1.60	−8.31	−7.73	−5.06	*3.25*	−9.65	−4.65	−0.01	*9.64*
9	1.41	1.62	−8.95	−7.89	−6.51	*2.44*	−2.90	−0.04	1.59	*4.49*
16	1.69	1.79	−12.08	−11.25	−7.43	*4.65*	−13.96	−7.13	−0.02	*13.95*
3	3.40	3.58	−39.78	−39.72	−39.60	*0.18*	−38.59	−38.57	−38.56	*0.03*
10	4.36	4.27	−54.04	−50.50	−47.33	*6.71*	−51.24	−0.19	27.02	*78.26*
4	6.05	6.22	−59.73	−59.55	−59.37	*0.36*	−46.48	−6.25	−3.29	*43.19*

Table 1 also includes two indicators used to assess each frame's sensitivity to instability. The first is an amplification factor proposed by Merchant [14] to estimate second-order forces from first-order LA results. Merchant's amplifier is based on a frame's critical buckling load factor, α_cr_, as follows:

(1) $$AF_{\alpha_{cr}}=\frac{1}{1-1\;/\;\alpha_{cr}}$$

**Fig. 3:**
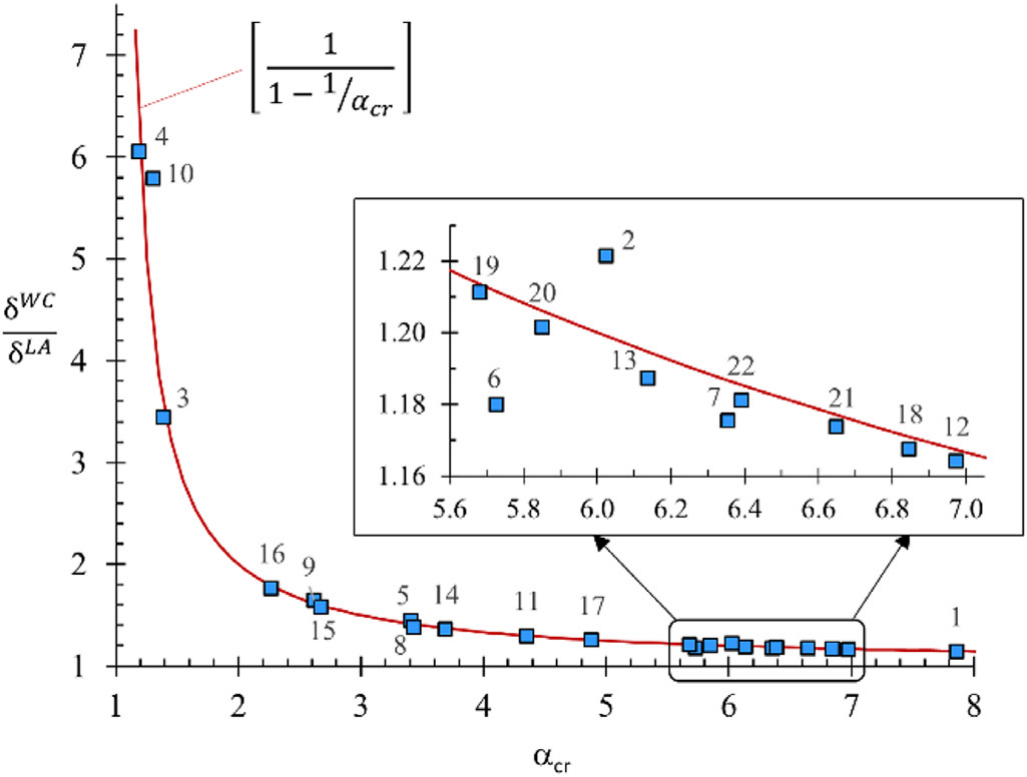
Plot of the maximum ratio of second- to first-order lateral displacements at any joint in each benchmark frame, shown together with function representing Merchant's amplification factor [14].

The second indicator is the maximum ratio of second-order (WC) to first-order (LA) lateral joint displacements at any joint in the frame, or $\left(\delta^{WC}\;/\;\delta^{LA}\right)$. A comparison of the two indicators is presented in Fig. 3.

The Mendeley data repository [3] includes an Excel data file with a tab for each benchmark frame. Each tab lists the lateral displacements of every joint and the design moments (maximum moment) in every member in the frame, as simulated by WC, SIPC, and LA analyses for both imperfection (and lateral loading) directions; for the load cases investigated. This file also provides the critical buckling load ratio, α_cr_, for each frame, corresponding to the lowest sway buckling mode of the structure, as determined in an elastic eigenvalue buckling analysis (LBA) within MASTAN2.

## 2 Experimental Design, Materials and Methods

The finite element models of the benchmark frames were created in MASTAN2 as described in Table 2 below.

**Table 2:** Details of finite element modeling of benchmark frames.

**Finite element models of frames**	

Geometry	
Members	Modeled as 4 planar line (6 dof) elements
Multiple lean-on columns	Modeled as single column on each side of frame
Imperfections	Global sway; Magnitude: H/500; Direction: same as lateral load (or natural lean, for gravity-only frames). Imperfections were modeled by horizontally translating all nodes by an amount equal to their vertical coordinate divided by 500.

Material	
Steel	E = 200 GPa (29,000 ksi); F_y_ = 345 MPa (50 ksi) or 250 MPa (36 ksi);
Elastic modulus used	Stiffness reduced to 0.8E, per AISC's *direct analysis method* (DAM) [16], for all elastic analyses; Stiffness reduced to 0.9E for *geometric and material nonlinear analyses with imperfections* (GMNIA);
Yield strength used	F_y_ (for DAM), 0.9F_y_ (for GMNIA);

Loading	
Gravity load	Distributed loads modeled by equivalent concentrated loads applied at the beam quarter points. Gravity loading includes self-weight of components.
Lateral load	Applied as concentrated loads in direction as shown (Fig. 1)
Live/Dead load ratios	Equal to L/D of original frame in literature (where applicable)
LRFD load combinations [15]	Multi-story frames without wind: 1.2D + 1.6L + 0.5L_r_
	Single-story frames with and without wind: 1.2D + 1.6 L_r_ + (L or 0.5W)
	Single- and multi-story frames with wind: 1.2 D + 1.0L + 0.5L_r_ + 1.0W
Load application	In all analyses, gravity and lateral loads are applied proportionally (simultaneously)
Load magnitudes	Determined such that the applied load ratio ≈ 1.0 in GMNIA, using appropriate factored load combination and initial global sway imperfection in the direction of lateral loading or in direction shown in Fig. 1 (for gravity-only frames: 3, 9, and 10).

**Fig. 4:**
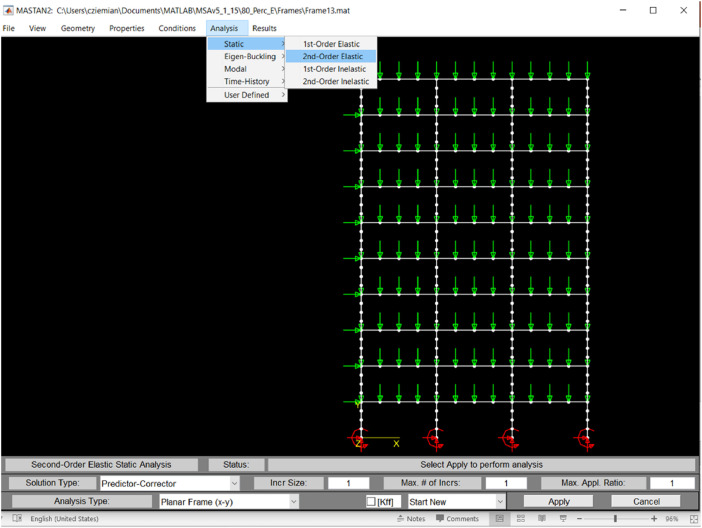
Illustration of MASTAN2 menu selections for SIPC analysis.

Several different analyses were performed on each frame model in order to validate the proposed SIPC method and examine its accuracy and precision as a function of the frame's sensitivity to second order effects. The parameter settings used for each analysis type are described in Table 3. Fig. 4 also provides an illustration of how the analysis options are selected within MASTAN2.

**Table 3:** Details of the computational analyses performed in MASTAN2 on each frame.

**Frame analyses**	

Elastic, first-order	
Linear (eigenvalue) buckling analysis (LBA)	Used to compute critical buckling load ratio, α_cr_, corresponding to lowest sway buckling mode. Performed on perfect geometry, using stiffness 0.8E.

Linear analysis (LA)	Results used for computations of second- to first-order ratios and/or amplification factors. Performed on imperfect geometry, using stiffness 0.8E.

Elastic, second-order	
Note: MASTAN2 accounts for second-order effects using an updated Langrangian formulation and geometric stiffness matrices [13].	
Geometric nonlinear analysis with imperfections (GNIA)	Single increment predictor-corrector (SIPC) analysis, a proposed approximate GNIA method; Increment size = 1; Performed on imperfect geometry, using stiffness 0.8E.
GNIA	Work-control (WC) analysis with step-size = 0.001. Used as the ‘expected’ or ‘exact’ elastic solution for comparisons when assessing the accuracy of the SIPC method; Performed on imperfect geometry, using stiffness 0.8E.

Inelastic, second-order	
Geometric and material nonlinear analysis with imperfections (GMNIA)	Results used only to determine magnitude of loading placed on the frames; Performed using modified tangent stiffness method [1] with imperfect geometry, stiffness 0.9E, and yield stress 0.9F_y_; Objective is to have structure fail at 1.0*load factor.

The described error analysis is based on comparisons of the lateral displacement of each joint and the design moment in each member in the frame, as simulated by SIPC and WC analyses. As described in [1], the WC data was first filtered in order to avoid error calculations on extremely small and insignificant joint displacements and/or member design moments. Specifically, a joint displacement that was less than 1/1000 of the minimum member length in the frame was removed from consideration. Similarly, each member moment was first divided by the product of the member's plastic section modulus and material yield strength, resulting in the associated proportion of the member's plastic moment capacity. These normalized moments from the WC data were then filtered, for the same reasons described above. Specifically, a normalized member moment that was less than 0.10, or 10% of the amount of moment required to form a flexural plastic hinge in the member, was removed from consideration.

All error analyses were based on percent error calculations. For example, the comparison of the lateral displacements of joint *j* was made by computing the percent error as,

(2) $$\epsilon_{j}=100*\frac{\left(\delta_{j}^{SIPC}-\delta_{j}^{WC}\right)}{\delta_{j}^{WC}}\;$$

## Ethics Statement

The authors did not use any animal or human subjects, and did not use data from social media platforms. This article conforms to Elsevier's standards of ethical publishing.

## CRediT Author Statement

**Constance W. Ziemian** Methodology, Software, Formal analysis, Investigation, Data curation, Writing – original draft, Writing – review & editing, Visualization; **Ronald D. Ziemian** Conceptualization, Methodology, Software, Validation, Investigation, Writing – review & editing.

## Declaration of Competing Interest

The authors declare that they have no known competing financial interests or personal relationships that have or could be perceived to have influenced the work reported in this article.
